# Prelabor cesarean section: the role of advanced maternal age and associated factors

**DOI:** 10.11606/s1518-8787.2021055002530

**Published:** 2021-04-05

**Authors:** Katrini Guidolini Martinelli, Silvana Granado Nogueira da Gama, André Henrique do Vale de Almeida, Marcos Nakamura-Pereira, Edson Theodoro dos Santos

**Affiliations:** I Universidade Federal do Espírito Santo Programa de Pós-Graduação em Saúde Coletiva VitóriaES Brasil Universidade Federal do Espírito Santo. Programa de Pós-Graduação em Saúde Coletiva. Vitória, ES, Brasil; II Fundação Oswaldo Cruz Escola Nacional de Saúde Pública Departamento de Métodos Quantitativos Rio de JaneiroRJ Brasil Fundação Oswaldo Cruz. Escola Nacional de Saúde Pública. Departamento de Métodos Quantitativos. Rio de Janeiro, RJ, Brasil; III Universidade Estadual de Feira de Santana Departamento de Saúde Feira de SantanaBA Brasil Universidade Estadual de Feira de Santana. Departamento de Saúde. Feira de Santana, BA, Brasil; IV Fundação Oswaldo Cruz Instituto Fernandes Figueira Rio de JaneiroRJ Brasil Fundação Oswaldo Cruz. Instituto Fernandes Figueira. Rio de Janeiro, RJ, Brasil; V Universidade Federal do Espírito Santo Departamento de Medicina Social VitóriaES Brasil Universidade Federal do Espírito Santo. Departamento de Medicina Social. Vitória, ES, Brasil

**Keywords:** Cesarean Section, Maternal and Child Health, Maternal Age, Pregnancy Complications

## Abstract

**OBJECTIVE:**

to evaluate whether advanced maternal age (AMA) is associated with prelabor cesarean section and to identify the factors associated with prelabor cesarean section in AMA women, according to the mode of type of labor financing (private or public).

**METHODS:**

Based on the Birth in Brazil survey, the research was conducted on representative sample of mothers for the country (Brazil), regions, type of hospital and location (capital or not), in 2011/2012. This study included 15,071 women from two age groups: 20–29 years and ≥ 35 years. The information was collected from interviews with puerperal woman, prenatal cards, and medical records of mothers and newborns. Multiple logistic regression modelling was used to verify the association between prelabor cesarean section and maternal, prenatal and childbirth characteristics, according to the mode of financing.

**RESULTS:**

Our results showed a higher use of prelabor cesarean section for AMA (≥ 35 years) women in the public service (OR = 1.63; 95%CI 1.38–1.94) and in the private service (OR = 1.44; 95%CI 1.13–1.83), compared with women aged 20–29 years. In the adjusted model, we recorded three factors associated with the prelabor cesarean section in AMA women in both, public and private sectors: the same professional in prenatal care and childbirth (OR = 4.97 and OR = 4.66); nulliparity (OR = 6.17 and OR = 10.08), and multiparity with previous cesarean section (from OR = 5.73 to OR = 32.29). The presence of obstetric risk (OR = 1.94; 95%CI .44–2.62) also contributed to the occurrence of prelabor cesarean section in women who gave birth in the public service.

**CONCLUSIONS:**

AMA was an independent risk factor for prelabor cesarean in public and private services. In the public, prelabor cesarean in AMA was more influenced by clinical criteria. Higher chance of prelabor cesarean section in nulliparous women increases the chance of cesarean section in multiparous women, as we showed in this study, which increases the risk of anomalous placental implantation.

## INTRODUCTION

The percentage of women with advanced maternal age (AMA) has been increasing in high^1^, middle-, and low-income countries^4,5^. In Brazil, in 1994, only 7.6% of births occurred in women aged 35 and older. In 2017 this percentage increased to 14.4%, indicating an increase of almost 90% in the number of pregnant women in this age group^5^. AMA has been associated with several negative maternal and perinatal outcomes, such as gestational diabetes, preeclampsia, placenta previa, placental abruption, preterm birth, low birth weight, congenital anomalies, and perinatal mortality^3,6,7^.

Some of these conditions related to AMA increase the chances of cesarean section or represent unequivocal indications for surgery—as is the case for total placenta previa. A 17 year-long study found a greater chance of cesarean section in both nulliparous and multiparous pregnancies among AMA women^8^. Another study has also showed an increase in the chance of elective and emergency cesarean sections in AMA women^9^.

Although women with AMA are more prone to cesarean section because of complications in pregnancy^3,10^, the strength of association remains even with a comprehensive adjustment for confounding factors that incorporate comorbidities before pregnancy, demographic and anthropometric factors, as well as pregnancy, obstetric, and fetal complications^8^.

According to the World Health Organization (WHO), cesarean section without medical indication may bring risks to maternal and perinatal health. These risks include admission to the Intensive Care Unit (ICU), need for blood transfusion, and fetal and neonatal mortality^11^. Elective cesarean section has also been associated with newborn respiratory disorders, especially when performed prior to 39 weeks^12^.

In addition to AMA, the mode of financing of the childbirth has a considerable influence on the rate of cesarean section^13^. This is evidenced in Brazil, where around 85% of all newborn in the private sector are delivered via cesarean section, compared with 35% in the public sector^14^. This study aims to evaluate whether AMA is associated with prelabor cesarean section. It also aims to identify the factors associated with prelabor cesarean section in AMA women, according to the mode of financing the childbirth (private or public).

## METHODS

Data collection occurred during the period from February 2011 to October 2012. The study draws on a national hospital-based survey of puerperal women and their newborns, Birth in Brazil. The sample was selected in three stages. The first stage included hospitals with more than 500 deliveries per year, stratified according to the Brazil’s macro-regions (North, South, Northeast, Southeast, and Midwest), location (capital or other cities) and type of service (public, private or mixed). In the second stage, using the reverse sampling method, researchers defined the number of days necessary to interview 90 puerperal women in each one of the 266 previously selected hospitals (minimum of seven days). In the third stage, the puerperal women and their newborns were selected. Additional information on the sample design can be found in the reference study^16^.

“Birth in Brazil” collected data from electronic questionnaires, interviews with puerperal woman during hospital stay, prenatal cards photographed and transcribed into a standardized form, and maternal records. Medical records were analyzed after the patient’s discharge or any time before the 42nd day of hospitalization. Further details on data collection are described elsewhere^16^.

Although the data were collected from 2011 to 2012, there was little change in relation to this topic in Brazil. The rate of cesarean section remains high (51.8% in 2012 and 54.1% in 2017), although the rate of prelabor cesarean section has slightly decreased in the period, according to the study by Leal et al. (2019)^17^.

This analysis considered all puerperal women aged 20–29 years and 35 years or older, present in the sample “Birth in Brazil” to fulfill de objective of this study. The cutoff point of the AMA group was chosen based on the Brazilian neonatal mortality rate that exceeds seven deaths per 1,000 live births and begins to advance faster for women aged 35 years or older^18^. The age group of 20–29 years is considered medically favorable for reproduction and childbirth^19^.

The first variable, maternal age, was divided into two groups: 20–29 years and ≥ 35 years of age. The birth outcome variable was related to the type of childbirth according to labor (vaginal, forceps-assisted, prelabor cesarean section, and labor with cesarean section). For statistical analysis, we used a single category for childbirths in which women went into labor (vaginal, forceps-assisted, and cesarean after spontaneous, or induced labor). Women undergoing prelabor cesarean section composed the second category. Furthermore, we used mode of financing of childbirth to stratify the results – mode of service where child was delivered (public or private). Researchers collected data on maternal age and financing of childbirth via interview, whereas the outcome variable was collected based on maternal record.

Additional variables related to prenatal care and childbirth: same health professional who performed prenatal care and childbirth (yes/no), the need to search for a place to give birth – in cases where the mother was not assisted at the first maternity hospital sought for childbirth (yes/no), initial preference for the type of childbirth (cesarean section, vaginal/no preference), minimum overall adequacy of prenatal care (adequate, inadequate), and obstetric risk (low and high). The obstetric backgrounds addressed included: the number of previous cesarean sections (none, one, and two or more), and parity (nulliparous, one or two previous childbirth, and three or more previous childbirth).

The minimum Adequacy of Prenatal Care Utilization Index recommended by the Brazilian Ministry of Health was adopted and adapted by Domingues et al.^20^. Prenatal care was considered minimally adequate when the onset of care occurred before the 12th week of gestation; when the number of appointments was appropriate for the gestational age at delivery; when at least one of the routine tests was carried out (serology for syphilis, fasting glucose blood test, urine test, HIV serology, and ultrasonography); and when the pregnant women has received guidance regarding the maternity hospital for delivery.

Regarding obstetric risk, women who met the following criteria were considered low risk: no diabetes or hypertensive disease, no obesity (BMI < 30 kg/m^2^), HIV negative, infant gestational age between 37 and 41 weeks at birth, single pregnancy, fetus in cephalic presentation, birth weight between 2,500g and 4,499g, and birth weight between the 5th and 95th percentile weight according to gestational age. These favorable outcomes for both mother and newborn were considered proxy for a pregnancy without complications. Women who presented pathologies that did not fall into these groups were not included in the criterion^21^. Finally, obstetric risk was represented by a summary measure (low and high).

The study considered the following sociodemographic characteristics: maternal age (20–29 years and ≥ 35 years – AMA), mother’s level of education (≤ 7 years, 8–10 years, ≥ 11 years), skin color (White and non-white) and marital status (living with or without a partner).

All analyses considered the complex design of sampling and they were carried out according to the mode of financing of childbirth (public or private). Moreover, each stratum received a calibration procedure by basic sample weights to ensure that the distribution of puerperal women was comparable to that observed in births of the population sampled in 2011.

The Rao-Scott chi-square test with 95% confidence interval was uses in order to test the homogeneity of the proportions of the maternal, prenatal and childbirth characteristics according to the maternal age (20–29 years and 35 years of age or older). The same test was used to test whether these characteristics were associated with prelabor cesarean sections. Subsequently, to verify the association of AMA and prelabor cesarean sections, we performed a logistic regression model. We included controls for confounding factors for each type of financing. Additionally, we applied a logistic regression model for AMA women – according to the mode of financing – to identify factors associated with prelabor cesarean section. We tested the effects for interaction before the final analysis in all regressions, and when they were present, they were maintained in the final analyses. Pseudo-R^2^statistics (Cox & Snell and Nagelkerke) was used to choose the best model, whose value was closer to 1. Adjusted analyses included all variables from the unadjusted analysis with p-value < 0.10.

This research was approved by the Research Ethics Committee of the National School of Public Health of the Oswaldo Cruz Foundation, No. 92/2010 and 2.041.963/2017. The digital consent of each puerperal woman was obtained with an informed consent form, which was issued before the interview. The same applies for the hospital units directors.

## RESULTS

We included 15,071 women in this analysis. Out of this group, 12,562 were aged from 20 to 29 years (83.4%), 2,043 were aged from 35 to 39 years (13.5%) and 466 were older than 40 years (3.1%). Among women who had public funding for childbirth, 13.6% were AMA, whereas in private facilities, 28.4% of women who gave birth were AMA.

In the public sector, AMA women presented lower levels of education, lived with a partner more frequently, were more frequently served by the same physician both during prenatal care and childbirth, searched less for the right health care unit for childbirth, had higher preference for cesarean sections, showed higher parity, and had more previous cesarean sections and more prelabor cesarean sections. Regarding obstetric risk, all indicators assessed had a greater prevalence among AMA women, except for HIV infection (Table 1).

**Table 1:** Maternal, prenatal and childbirth characteristics, by maternal age and type of financing of childbirth, Brazil, 2011–2012.

Variables^a^	Public healthcare		χ^2^ p	Private healthcare		χ^2^ p
	20–29 y.old (n = 10,364)	≥ 35 y.old (n = 1,636)		20-29 y.old (n = 2,198)	≥ 35 y.old (n = 873)	
	n (%)	n (%)		n (%)	n (%)	
Maternal characteristics						
Maternal schooling			< 0.0001			0.023
≤ 7 years	2,714 (26.3)	716 (44.0)		58 (2.7)	35 (4.0)	
8–10 years	2,908 (28.2)	284 (17.5)		224 (10.3)	60 (7.0)	
≥ 11 years	4,704 (45.6)	627 (38.5)		1,898 (87.0)	771 (89.0)	
Skin color			0.456			< 0.0001
White	7,367 (71.1)	1,141 (69.7)		1,070 (48.7)	347 (39.7)	
non-white	2,996 (28.9)	495 (30.3)		1,127 (51.3)	527 (60.3)	
Marital status			0.008			0.090
With partner	1,951 (18.9)	247 (15.1)		263 (12.0)	76 (8.7)	
Without a partner	8,387 (81.1)	1,387 (84.9)		1,933 (88.0)	797 (91.3)	
Prenatal and childbirth characteristics						
Same doctor PN and childbirth			0.002			< 0.0001
Yes	999 (9.7)	209 (12.8)		1,657 (75.5)	744 (85.1)	
No	9,343 (90.3)	1,427 (87.2)		538 (24.5)	130 (14.9)	
Adequacy PN			0.621			0.006
Inadequate	8,760 (84.5)	1,393 (85.1)		1,785 (81.2)	746 (85.5)	
Adequate	1,603 (15.5)	243 (14.9)		412 (18.8)	127 (14.5)	
Search for healthcare unit for childbirth			0.004			0.516
No	7,919 (76.5)	1,325 (81.0)		2,072 (94.3)	830 (95.1)	
Yes	2,438 (23.5)	310 (19.0)		125 (5.7)	43 (4.9)	
Initial preference for the type of childbirth			< 0.0001			< 0.0001
Vaginal/no preference	8,052 (77.7)	1,065 (65.1)		1,283 (58.4)	396 (45.4)	
Cesarean	2,310 (22.3)	570 (34.9)		914 (41.6)	477 (54.6)	
Type of childbirth			< 0.0001			< 0.0001
Vaginal/Forceps	5,870 (58.2)	778 (48.8)		282 (13.0)	73 (8.4)	
Prelabor cesarean	3,590 (35.6)	723 (45.3)		1,805 (83.3)	773 (89.3)	
Cesarean with labor	626 (6.2)	93 (5.8)		81 (3.7)	20 (2.3)	
Parity			< 0.0001			< 0.0001
Nulliparous	4,255 (41.1)	213 (13.0)		1,443 (65.7)	294 (33.6)	
1 to 2 previous deliveries	5,067 (48.9)	818 (50.0)		730 (33.2)	546 (62.5)	
3 or more previous deliveries	1,401 (10.0)	604 (36.9)		24 (1.1)	34 (3.9)	
Previous cesarean sections			< 0.0001			< 0.0001
None	4,010 (38.7)	921 (56.3)		290 (13.2)	146 (16.7)	
Nulliparous	4,255 (41.1)	213 (13.0)		1,443 (65.7)	294 (33.7)	
One	1,558 (15.0)	337 (20.6)		417 (19.0)	341 (39.1)	
Two or more	541 (5.2)	164 (10.0)		48 (2.2)	92 (10.5)	
Composition of obstetric risk						
Hypertensive disease^b^	1,000 (9.6)	329 (20.1)	< 0.0001	255 (11.6)	149 (17.0)	0.002
Pre-pregnancy diabetes	94 (0.9)	48 (2.9)	< 0.0001	08 (0.4)	22 (2.5)	< 0.0001
Pregnancy diabetes	734 (7.1)	265 (16.2)	< 0.0001	162 (7.4)	109 (12.5)	< 0.0001
BMI ≥ 30Kg/m^2^	885 (8.5)	259 (15.8)	< 0.0001	215 (9.8)	102 (11.7)	0.258
HIV-positive	52 (0.5)	14 (0.9)	0.122	02 (0.1)	01 (0.1)	0.559
GA < 37 or > 41 weeks	1,271 (12.3)	271 (16.6)	< 0.0001	261 (11.9)	104 (11.9)	0.917
Multiple pregnancy	86 (0.8)	32 (2.0)	0.003	48 (2.2)	21 (2.4)	0.647
Non cephalic presentation	383 (3.7)	118 (7.2)	< 0.0001	96 (4.4)	49 (5.6)	0.122
Weight < 2500 / > 4499 g	841 (8.1)	249 (15.2)	< 0.0001	182 (8.3)	77 (8.8)	0.778
Weight per GA < 5º e > 95º	1,108 (10.7)	243 (14.9)	< 0.0001	215 (9.8)	94 (10.8)	0.394
Summary measure of obstetric risk			< 0.0001			0.005
Low	6,052 (58.4)	664 (40.6)		1,274 (58.0)	442 (50.6)	
High	4,312 (41.6)	971 (59.4)		923 (42.0)	431 (49.4)	

PN: prenatal care; GA: gestational age; BMI: body mass index.

^a^ Some variables present missing data.

^b^ Hypertensive disease: chronic hypertension, pre-eclampsia, eclampsia or syndrome HELLP.

Furthermore, in the private sector, AMA women presented higher levels of education, were predominantly white, were served by the same physician both during prenatal care and childbirth, had inadequate prenatal care, had more preference for cesarean sections, showed higher parity, had more previous cesarean sections, and had more prelabor cesarean sections. As for obstetric risk, hypertensive disease, pre-gestational and gestational diabetes were more frequent in AMA women, as well as the classification of gestational risk (Table 1).

Table 2 shows that AMA women received more prelabor cesarean sections than women aged from 20 to 29, both in public (45.4% vs. 35.6%) and private health care services (89.3% vs. 83.2%). In addition to AMA, other factors associated with prelabor cesarean sections included: higher levels of education, white skin color, same physician for both prenatal care and during childbirth, not searching for the right healthcare unit for childbirth, initial preference for cesarean section, nulliparity, previous cesarean sections, and risky pregnancy. As for the public service, an association was observed between adequate prenatal care and cesarean sections.

**Table 2:** Type of childbirth according to maternal and prenatal characteristics and childbirth by type of financing of childbirth, Brazil, 2011–2012.

Variables	Public healthcare		χ^2^ p	Private healthcare		χ^2^ p
	Vaginal/ Cesarean with labor (n = 7,367)	Prelabor cesarean (n = 4,313)		Vaginal/ Cesarean with labor (n = 457)	Prelabor cesarean (n = 2,578)	
	n (%)	n (%)		n (%)	n (%)	
Maternal characteristics						
Maternal age			< 0.0001			< 0.0001
20–29 years old	6,496 (64.4)	3,590 (35.6)		364 (16.8)	1,805 (83.2)	
≥ 35 years old	871 (54.6)	723 (45.4)		93 (10.7)	773 (89.3)	
Maternal schooling			< 0.0001			0.022
≤ 7 years	2,316 (69.0)	1,042 (31.0)		26 (28.0)	67 (72.0)	
8–10 years	2,028 (65.1)	1,085 (34.9)		51 (18.1)	231 (81.9)	
≥ 11 years	3,001 (58.1)	2,163 (41.9)		376 (14.3)	2,259 (85.7)	
Skin color			0.001			0.016
White	5,358 (64.7)	2,922 (35.3)		235 (16.7)	1,169 (83.3)	
non-white	2,008 (59.1)	1,392 (40.9)		222 (13.6)	1,409 (86.4)	
Prenatal and childbirth characteristics						
Same doctor PN and childbirth			< 0.0001			< 0.0001
Yes	344 (29.3)	832 (70.7)		224 (9.4)	2,153 (90.6)	
No	7,011 (66.8)	3,477 (33.2)		231 (35.3)	423 (64.7)	
Adequacy PN			< 0.0001			0.199
Inadequate	6,344 (64.2)	3,541 (35.8)		359 (14.3)	2,148 (85.7)	
Adequate	1,023 (57.0)	772 (43.0)		97 (18.4)	430 (81.6)	
Search for healthcare unit for childbirth			< 0.0001			0.001
Yes	1,852 (69.7)	804 (30.3)		44 (26.5)	122 (73.5)	
No	5,513 (61.1)	3,506 (38.9)		413 (14.4)	2,455 (85.6)	
Summary measure of obstetric risk			< 0.0001			< 0.0001
Low	4,534 (69.4)	1,996 (30.6)		290 (17.1)	1,404 (82.9)	
High	2,832 (55.0)	2,318 (45.0)		166 (12.4)	1,174 (87.6)	
Initial preference for the type of childbirth			< 0.0001			< 0.0001
Vaginal / no preference	6,138 (69.2)	2,730 (30.8)		389 (23.5)	1,263 (76.5)	
Cesarean	1,228 (43.7)	1,583 (56.3)		68 (4.9)	1,315 (95.1)	
Parity			< 0.0001			< 0.0001
Multipara without previous CS	4,116 (84.8)	737 (15.2)		205 (47.8)	224 (52.2)	
Nulliparous	2,541 (58.9)	1,773 (41.1)		219 (12.8)	1,491 (87.2)	
Multipara with previous CS = 1	636 (35.0)	1,180 (65.0)		29 (3.8)	726 (96.2)	
Multipara with previous CS ≥ 2	73 (10.5)	623 (89.5)		04 (2.9)	136 (97.1)	

CS: cesarean section; PN: prenatal care.

Table 3 presents the adjusted model of factors associated with prelabor cesarean sections in both public and private health care. Maternal age ≥ 35 years increases the likelihood of prelabor cesarean section by roughly 50%, when compared to postpartum women aged 20–29 years. The factors that yielded the greatest increase in the chance of cesarean section without labor included: previous cesarean section, nulliparity and the same medical doctor during both prenatal care and childbirth, both in public and private health care services.

**Table 3:** Factors associated with cesarean section without labor by type of financing of labor, Brazil, 2011–2012.

Variables	Public healthcare			Private healthcare		
	OR (95%CI)	Model 1^a^ AOR (95%CI)	Model 2^b^ AOR (95%CI)	OR (95%CI)	Model 1^a^ AOR (95%CI)	Model 2^b^ AOR (95%CI)
Maternal characteristics						
Maternal age						
20–29 years old	1.00 (ref.)	1.00 (ref.)	1.00 (ref.)	1.00 (ref.)	1.00 (ref.)	1.00 (ref.)
≥ 35 years old	1.50 (1.32–1.70)	1.63 (1.37–1.93)	1.63 (1.38–1.94)	1.71 (1.34–2.17)	1.50 (1.19–1.89)	1.44 (1.13–1.83)
Maternal schooling						
≤ 7 years	1.00 (ref.)	1.00 (ref.)	1.00 (ref.)	1.00 (ref.)	1.00 (ref.)	-
8–10 years	1.19 (1.02–1.39)	1.20 (0.99–1.44)	1.20 (0.99–1.45)	1.76 (1.01–3.09)	1.62 (0.90–2.90)	-
≥ 11 years	1.61 (1.37–1.90)	1.37 (1.12–1.67)	1.38 (1.13–1.68)	2.37 (1.26–4.43)	1.27 (0.72–2.23)	-
Skin color						
White	1.00 (ref.)	1.00 (ref.)	-	1.00 (ref.)	1.00 (ref.)	-
non-white	1.29 (1.11–1.49)	1.06 (0.92–1.22)	-	1.28 (1.05–1.56)	0.83 (0.62–1.20)	-
Prenatal and childbirth characteristics						
Same doctor PN and childbirth						
No	1.00 (ref.)	1.00 (ref.)	1.00 (ref.)	1.00 (ref.)	1.00 (ref.)	1.00 (ref.)
Yes	4.90 (3.95–6.08)	4.55 (3.69–5.62)	4.57 (3.71–5.63)	5.26 (3.44–8.06)	4.14 (2.61–6.57)	4.18 (2.66–6.59)
Adequacy PN						
Inadequate	1.00 (ref.)	1.00 (ref.)	1.00 (ref.)	-	-	-
Adequate	1.34 (1.16–1.56)	1.26 (1.06–1.49)	1.26 (1.07–1.49)	-	-	-
Search for healthcare unit for childbirth						
Yes	1.00 (ref.)	1.00 (ref.)	1.00 (ref.)	1.00 (ref.)	1.00 (ref.)	-
No	1.48 (1.25–1.74)	1.31 (1.11–1.55)	1.32 (1.11–1.56)	2.12 (1.35–3.32)	1.34 (0.82–2.17)	-
Summary measure of obstetric risk						
Low	1.00 (ref.)	1.00 (ref.)	1.00 (ref.)	1.00 (ref.)	1.00 (ref.)	1.00 (ref.)
High	1.87 (1.67–2.09)	2.08 (1.82–2.37)	2.08 (1.82–2.37)	1.50 (1.22–1.83)	1.62 (1.22–2.13)	1.59 (1.21–2.08)
Initial preference for the type of childbirth						
Vaginal / no preference	1.00 (ref.)	1.00 (ref.)	1.00 (ref.)	1.00 (ref.)	1.00 (ref.)	1.00 (ref.)
Cesarean	2.90 (2.54–3.30)	1.56 (1.33–1.83)	1.57 (1.34–1.84)	6.03 (4.52–8.05)	3.84 (2.80–5.27)	3.84 (2.79–5.29)
Parity						
Multipara without previous CS	1.00 (ref.)	1.00 (ref.)	1.00 (ref.)	1.00 (ref.)	1.00 (ref.)	1.00 (ref.)
Nulliparous	3.95 (3.45–4.51)	4.03 (3.41–4.77)	4.03 (3.41–4.77)	6.24 (4.44–8.77)	6.24 (4.24–9.19)	5.94 (4.15–8.51)
Multipara with previous CS = 1	10.53 (8.61–12.89)	9.78 (7.88–12.14)	9.79 (7.89–12.15)	22.96 (15.78–33.42)	12.92 (9.03–18.47)	12.41 (8.73–17.64)
Multipara with previous CS ≥ 2	47.59 (32.56–69.55)	46.50 (30.7–70.4)	46.55 (30.8–70.4)	30.54 (9.39–99.39)	11.69 (3.38–40.37)	11.34 (3.42–37.63)

CS: cesarean section; PN: prenatal care; ref.: reference.

^a^ Model adjusted for all variables included in the gross analysis.

^b^ Model adjusted only by the variables that remained in the final model.

Interaction was tested by the same doctor during the PN and childbirth*initial preference through childbirth; adequacy of the PN*same professional during the PN and childbirth; age*schooling; education*skin color; adequacy of the obstetric PN*obstetric risk.

Table 4 shows the factors associated with prelabor cesarean section in AMA women. In both sectors, women with higher levels of education, who were assisted by the same medical doctor both in prenatal care and during childbirth, preferred cesarean section, were nulliparous, and had previous cesarean section, were more likely to receive prelabor cesarean section. White skin color and high obstetric risk were associated with prelabor cesarean section only in the public healthcare service.

**Table 4:** Type of childbirth according to maternal and prenatal characteristics and childbirth by type of financing of childbirth in older women ≥ 35 years (n = 2,460), Brazil, 2011–2012.

Variables	Public healthcare		χ^2^ p	Private healthcare		χ^2^ p
	Vaginal/ Cesarean with labor (n = 871)	Prelabor cesarean (n = 723)		Vaginal/ Cesarean with labor (n = 93)	Prelabor cesarean (n = 773)	
	n (%)	n (%)		n (%)	n (%)	
Maternal characteristics						
Maternal schooling			< 0.0001			0.007
≤ 7 years	437 (62.6)	261 (37.4)		09 (25.7)	26 (74.3)	
8–10 years	145 (51.8)	135 (48.2)		07 (11.7)	53 (88.3)	
≥ 11 years	286 (46.8)	325 (53.2)		76 (10.0)	687 (90.0)	
Skin color			0.014			0.352
White	635 (57.2)	476 (42.8)		43 (12.5)	302 (87.5)	
non-white	236 (48.8)	248 (51.2)		50 (9.6)	472 (90.4)	
Prenatal and childbirth characteristics						
Same doctor PN and childbirth			< 0.0001			< 0.0001
No	831 (59.7)	560 (40.3)		45 (34.9)	84 (65.1)	
Yes	40 (19.6)	164 (80.4)		47 (6.4)	690 (93.6)	
Adequacy PN			0.113			0.494
Inadequate	754 (55.7)	600 (44.3)		77 (10.4)	666 (89.6)	
Adequate	117 (48.8)	123 (51.2)		15 (12.3)	107 (87.7)	
Search for healthcare unit for childbirth			< 0.0001			0.024
Yes	202 (66.7)	101 (33.3)		09 (20.9)	34 (79.1)	
No	669 (51.8)	623 (48.2)		83 (10.1)	739 (89.9)	
Summary measure of obstetric risk			0.001			0.658
Low	396 (61.7)	246 (38.3)		48 (10.9)	392 (89.1)	
High	475 (49.8)	478 (50.2)		45 (10.6)	381 (89.4)	
Initial preference for the type of childbirth			< 0.0001			< 0.0001
Vaginal / no preference	654 (62.9)	385 (37.1)		70 (17.9)	320 (82.1)	
Cesarean	217 (39.0)	339 (61.0)		22 (4.6)	453 (95.4)	
Parity			< 0.0001			< 0.0001
Multipara without previous CS	684 (75.9)	217 (24.1)		63 (44.1)	80 (55.9)	
Nulliparous	68 (32,5)	141 (67,5)		16 (5,5)	274 (94,5)	
Multipara with previous CS = 1	107 (33,3)	214 (66,7)		11 (3,2)	331 (96,8)	
Multipara with previous CS ≥ 2	12 (7,3)	152 (92,7)		03 (3,3)	89 (96,7)	

CS: cesarean section; PN: prenatal care.

In the adjusted model, the factors associated with prelabor cesarean section in AMA women, in both public and private health care sectors, included: the same physician during both prenatal care and childbirth, initial preference for a cesarean section, nulliparity, one previous cesarean section, and two or more previous cesarean sections. In addition, for women who received attention in the public healthcare service, results indicated that the presence of obstetric risk contributed to the occurrence of prelabor cesarean sections (Table 5).

**Table 5:** Factors associated with cesarean section without labor in women aged ≥ 35 years (n = 2,460), by type of financing of labor, Brazil, 2011–2012.

Variables	Public Healthcare			Private Healthcare		
	OR (IC 95%)	Model 1^a^ AOR (IC 95%)	Model 2^b^ AOR (IC 95%)	OR (IC 95%)	Model 1^a^ AOR (IC 95%)	Model 2^b^ AOR (IC 95%)
Maternal Characteristics						
Maternal schooling						
≤ 7 years	1.00 (ref.)	1.00 (ref.)	-	1.00 (ref.)	1.00 (ref.)	1.00 (ref.)
8–10 years	1.55 (1.07–2.24)	1.21 (0.78–1.87)	-	2.93 (1.08–7.94)	3.39 (1.18–9.72)	3.42 (1.18–9.92)
≥ 11 years	1.92 (1.41–2.63)	1.29 (0.89–1.84)	-	3.42 (1.49–7.84)	1.42 (0.65–3.08)	1.42 (0.65–3.09)
Skin color						
White	1.00 (ref.)	1.00 (ref.)	-	-	-	-
non-white	1.42 (1.07–1.87)	1.01 (0.72–1.42)	-	-	-	-
Prenatal and Childbirth Characteristics						
Same doctor PN and childbirth						
No	1.00 (ref.)	1.00 (ref.)	1.00 (ref.)	1.00 (ref.)	1.00 (ref.)	1.00 (ref.)
Yes	6.03 (3.98–9.16)	4.67 (2.67–8.18)	4.97 (2.86–8.63)	8.33 (4.20–16.54)	4.73 (2.25–9.91)	4.66 (2.10–10.34)
Search for healthcare unit for childbirth						
Yes	1.00 (ref.)	1.00 (ref.)	-	1.00 (ref.)	1.00 (ref.)	-
No	1.85 (1.35–2.53)	1.31 (0.91–1.88)	-	2.55 (1.10–5.92)	0.93 (0.28–3.08)	-
Classification obstetric risk						
Low	1.00 (ref.)	1.00 (ref.)	1.00 (ref.)	-	-	-
High	1.61 (1.22–2.12)	1.97 (1.45–2.66)	1.94 (1.44–2.62)	-	-	-
Initial preference for the type of childbirth						
Vaginal / no preference	1.00 (ref.)	1.00 (ref.)	1.00 (ref.)	1.00 (ref.)	1.00 (ref.)	1.00 (ref.)
Cesarean	2.60 (2.05–3.30)	1.63 (1.21–2.19)	1.63 (1.22–2.19)	4.27 (2.43–7.51)	2.39 (1.12–5.12)	2.40 (1.11–5.17)
Parity						
Multipara without previous CS	1.00 (ref.)	1.00 (ref.)	1.00 (ref.)	1.00 (ref.)	1.00 (ref.)	1.00 (ref.)
Nulliparous	6.57 (4.28–10.09)	5.53 (3.47–8.80)	6.17 (3.93–9.67)	13.32 (6.46–27.48)	10.01 (4.53–22.48)	10.08 (4.50–22.60)
Multipara with previous CS = 1	6.52 (4.51–9.42)	5.49 (3.57–8.44)	5.73 (3.80–8.65)	23.93 (9.6–59.1)	12.97 (5.46–30.80)	12.97 (5.48–30.7)
Multipara with previous CS ≥ 2	40.82 (19.83–84.04)	31.34 (14.67–66.96)	32.29 (15.1–68.9)	19.98 (5.6–70.8)	9.47 (2.28–39.36)	9.43 (2.27–39.1)

CS: cesarean section; PN: prenatal care; ref.: reference.

^a^ Model adjusted for all variables included in the gross analysis.

^b^ Model adjusted only by the variables that remained in the final model.

Interaction was tested for: the same professional during the PN and childbirth*initial preference through childbirth, adequacy of the PN*same professional during the PN and childbirth, age*education schooling, education schooling*skin color, adequacy of the PN*obstetric risk.

## DISCUSSION

AMA women with private care presented more favorable maternal characteristics than those who gave birth in public health care services. These characteristics included higher education levels, less time searching for a place to give birth, and less obstetric risk. Regardless of the type of financing of childbirth, AMA was independently associated with prelabor cesarean section. Moreover, among advanced maternal age women, obstetric characteristics such as previous cesarean section or nulliparity, follow-up with a single physician during prenatal care and childbirth, and preference for cesarean section at the beginning of pregnancy, composed key factors for the occurrence of prelabor cesarean section. Furthermore, AMA women in public health care service also presented obstetric risk associated with prelabor cesarean section. Previous cesarean section, prenatal care, and delivery with the same physician were found as characteristics that contributed the most to a prelabor cesarean section.

Advanced maternal age women attended in public health care service presented similar characteristics to those of middle- and low-income women from other countries. These included: low schooling^4,22^, having a partner^4,22^, and multiparity^22^. On the other hand, women who attended the private health care service presented similar characteristics to those of high-income countries, such as high schooling^6^, white skin color^1^, nulliparity, and previous cesarean sections^2^. These aspects indicate an inequality of resources in Brazil. However, regardless of the type of financing of childbirth, postpartum AMA women had a higher percentage of prelabor cesarean sections when compared to younger women. This evidence agrees with results from other studies^2,4,23^.

As previously observed in similar analyses, the percentage of elective cesarean sections in Brazil is much higher than in other countries^24^. In Norway^23^, for example, the percentage of elective cesarean sections in advanced maternal age women was 18.0% for nulliparous women and 19.3% for multiparous women. These values are similar to those found in the WHO study conducted predominantly in low- and middle-income countries^4^. Conversely, in Brazil, this value reached 89.3% of AMA women in the private health care sector. However, the OR (1.63 in the public sector and 1.44 in the private sector) was even lower than that found for nulliparous women in Norway^23^, denoting the very high percentage of antepartum cesarean sections for young women in Brazil.

In a case comparable to Brazil, a study carried out in Australia showed that more AMA women were attended in private health care services (50.2%) than in public health care services (24.2%). In Australia, the percentage of advance maternal age women may have been higher because of the characteristics of the study, which only included nulliparous women who presented better socioeconomic conditions than multiparous women of advanced maternal age^3,25^. This non-clinical characteristic contributed to an increase in the probability of obstetric intervention, such as prelabor cesarean section, despite the fact that women who were attended in the private sector presented a lower likelihood of gestational complications^25^, as in our study.

The higher risk of cesarean section in women with advanced maternal age has been associated, by some authors, with a higher risk of dystocia because of myometrial contractility is predisposed to inefficiency^10,23^. However, when assessing only the relationship between AMA and antepartum cesarean section, this confounder was removed from the analysis. Some studies have addressed this relationship, and it remains equal, even with a comprehensive adjustment for confounding factors^23,25^. Theses studies suggest that the association is maintained by non-clinical variables.

In this study, even after controlling significant confounding factors – such as gestational risk, parity with identification of previous cesarean sections, and schooling – advanced maternal age continued to be associated with prelabor cesarean sections. The relationship between maternal age and risk of obstetric interventions is not yet well understood. However, it is possible that obstetricians and pregnant women may consider cesarean sections safer, especially when women are nulliparous closer to the end of their reproductive life, or if they have been submitted to assisted reproduction^8,13^. Also, the perception of risk may lower the threshold for interventions among obstetric personnel, could lead to iatrogenic interventions^2^. Furthermore, even without scientific evidence, pregnancy in AMA women is culturally considered as risky. A study conducted in Iceland, including only low-risk nulliparous women, found an association between prelabor cesarean section and AMA^26^.

When analyzing exclusively AMA women, regardless of the type of financing of childbirth, the variables most strongly associated with prelabor cesarean section were: the preference of the woman for cesarean section at the beginning of pregnancy, prenatal and childbirth care provided by the same physician, nulliparity, and previous cesarean section. It is plausible that these variables are not related to any clinical indication. Rather, that they are related to cultural and personal factors and the organization of healthcare services, which compel women to have cesarean sections even if they have a regular-risk pregnancy^13^.

Studies suggest that AMA women are influenced by a lowered treatment threshold for interventions, impacting the choices made by women, because affect their confidence regarding their own abilities to give birth without interventions^8,27^. A Canadian study with 1,865 women found AMA women (35 years and older) were twice as likely to request cesarean section from their care provider during their pregnancy (OR = 1.91; 95%CI: 1.07 to 3.41) compared to women aged 25–29 years^27^.

Although most clinical guidelines allow vaginal birth after cesarean in women with previous cesarean sections, the elective repeated cesarean section is often performed by choice of the women and/or the provider^28^. A population-based study in Brazil has shown that even in low-risk women who are eligible for trial of labor after cesarean section, the repetitive cesarean rate is high in the country. This is probably a result of non-clinical reasons^29^.

Only women with clinical and obstetric indication should receive this type of surgery in order to avoid maternal and neonatal complication. As a result, obstetric risk should be the main determining factor for performing prelabor cesarean section^30^. However, this association was found only in public health care service. This shows that non-obstetric factors have been instrumental for performing prelabor cesarean sections in private health care services.

The failure to adopt protocols established by obstetric health teams generates the misconception that cesarean sections are more advantageous for the infant^14^, especially for advanced maternal age women. The scenario could be changed if scientific evidence indicating greater chance of neonatal complications when a cesarean section occurs before 39 weeks prior to the onset of labor was considered when devising public policy^12^. Professionals responsible for prenatal care must explain to women the risks of unnecessary surgery and the advantages of vaginal birth. Women have attested the great influence of the physician who assists them in their decision regarding the type of birth^14^.

Therefore, one of this study strengths is its analysis of prelabor cesarean sections (focusing on antepartum clinical indications and intrapartum indication for labor dystocia). Most existing studies address only the relationship between AMA and cesarean sections, focusing mainly on clinical indications. Besides maternal age, this study analyzes women’s preference and the mode of financing the childbirth. It also considers the heterogeneous distribution of the type of labor in different health services, drawing on a representative sample from Brazil. On the other hand, the limitation of not having the variable “conception by in vitro fertilization” in order to analyze its association with the outcome is highlighted, although it is still not prevalent in Brazil, as it is not part of the procedures covered by the public sector or by health care plans, thereby being restricted to high-end private clinics.

## CONCLUSIONS

Prelabor cesarean section presents a complex scenario, especially for advanced maternal age women. It comprises not only clinical, cultural, social, organizational, and economic issues, but also the effect of maternal age on the type of childbirth. Therefore, the approach to AMA pregnancies should be multifaceted. This study advocates the application of both clinical (evaluation of vaginal birth after cesarean section, induction of labor) and non-clinical approaches (empowerment of pregnant women regarding the safer and healthier type of childbirth both for her and the baby, debunking mistaken beliefs, teamwork rather than individual work, face-to-face dialogues between pregnant women and health professionals about the lower chance of complications involved in vaginal birth). The risk of late pregnancy should be made clear to women who consider becoming pregnant at a later stage in life.

In the public health care service, women with advanced maternal age were influenced by clinical criteria in the choice of prelabor cesarean section. Whereas in private health care services, the organizational criteria stood out as key factors influencing women’s choices. Measures that stimulate vaginal birth should be adopted, especially in private health care services in order to reduce the high number of unnecessary cesarean sections. A change of attitude by health practitioners is also considered a necessary step to improve maternal health care for advanced maternal age women. Medical practitioners should base their actions on scientific evidence that indicates vaginal birth as the first choice. The principal aim should be lowering risks for mother and child during pregnancy and birth. Ultimately, this essay contests the claim that the organization of maternal healthcare services is a determinant of prelabor cesarean section. Rather, the chance of complications related to cesarean section are not properly outlined to AMA women prior to pregnancy in Brazil.
